# Water Deficit Transcriptomic Responses Differ in the Invasive *Tamarix chinensis* and *T. ramosissima* Established in the Southern and Northern United States

**DOI:** 10.3390/plants9010086

**Published:** 2020-01-09

**Authors:** Padmapriya Swaminathan, Michelle Ohrtman, Abigail Carinder, Anup Deuja, Cankun Wang, John Gaskin, Anne Fennell, Sharon Clay

**Affiliations:** 1 Agronomy, Horticulture and Plant Science, South Dakota State University, Brookings, SD 57007, USA; padmapriyaswaminathan@gmail.com (P.S.); mkohrtman@gmail.com (M.O.); carinderal5821@marybaldwin.edu (A.C.); adeuja25@gmail.com (A.D.); cankun.wang@osumc.edu (C.W.); 2 BioSystems Networks/Translational Research, South Dakota State University, Brookings, SD 57007, USA; 3 United States Department of Agriculture, Agricultural Research Service, Northern Plains Agricultural Research Laboratory, Sidney, MT 59270, USA; john.gaskin@usda.gov

**Keywords:** *Tamarix*, tamarisk, saltcedar, Tamaricaceae, RNA-Seq, water deficit, invasive species

## Abstract

*Tamarix* spp. (saltcedar) were introduced from Asia to the southern United States as windbreak and ornamental plants and have spread into natural areas. This study determined differential gene expression responses to water deficit (WD) in seedlings of *T. chinensis* and *T. ramosissima* from established invasive stands in New Mexico and Montana, respectively. A reference de novo transcriptome was developed using RNA sequences from WD and well-watered samples. Blast2GO analysis of the resulting 271,872 transcripts yielded 89,389 homologs. The reference *Tamarix* (Tamaricaceae, Carophyllales order) transcriptome showed homology with 14,247 predicted genes of the *Beta vulgaris* subsp. *vulgaris* (Amaranthaceae, Carophyllales order) genome assembly. *T. ramosissima* took longer to show water stress symptoms than *T. chinensis.* There were 2068 and 669 differentially expressed genes (DEG) in *T. chinensis and T. ramosissima,* respectively; 332 were DEG in common between the two species. Network analysis showed large biological process networks of similar gene content for each of the species under water deficit. Two distinct molecular function gene ontology networks (binding and transcription factor-related) encompassing multiple up-regulated transcription factors (MYB, NAC, and WRKY) and a cellular components network containing many down-regulated photosynthesis-related genes were identified in *T. chinensis*, in contrast to one small molecular function network in *T. ramosissima*.

## 1. Introduction

*Tamarix*, tamarisk, or saltcedar (*T. chinensis*, *T. ramosissima*, other *Tamarix* species, and hybrids) is an exotic shrub/tree that is the second most abundant riparian woody plant in the western United States [1]. Multiple introductions of *Tamarix* species were planted across the southwestern United States for erosion control and windbreak purposes [2]. This has led to hybridization, resulting in multiple complexes, rather than individual species, becoming the dominant *Tamarix* taxa in North America [3,4]. Once established, *Tamarix* is difficult to control and reinvasion commonly occurs unless plants are continually managed, often at great cost [5,6]. Economic and ecological consequences of *Tamarix* introduction are well documented [4,7], but little is currently known about genetic mechanisms that may contribute to invasion success.

*T. chinensis* and *T. ramosissima* are the most common invasive *Tamarix* in the United States. Extensive hybridization of these two species is found in North America, with hybrid genotypes extending from Oklahoma to Canada [4,8]. The genome size for *T. tetrandra* and *T. canariensis* is estimated at 1.6 pg and 1.53 pg, respectively [9]. Molecular phylogenies divide the *Tamarix* into eight clades, with *T. chinensis* and *T. ramosissima* being genetically distinct but clustering in the same clade [10,11,12]. In the United States, established southern *Tamarix* populations are dominated by *T. chinensis*, originally from forest zones of northern China, and are thought to establish most readily along river drainages [3,4,13]. *Tamarix* populations in the northern United States are dominated by *T. ramosissima*, originally found in middle Asian deserts under saline conditions. These genetic differences are supported by phenotypic differences along a latitudinal gradient. Plants successful in northern regions exhibit smaller stems, stem dieback, high energy investment in root material, truncated seed release periods, greater cold hardiness, and early-fall bud set and leaf senescence [13,14,15,16,17,18]. These responses suggest variation in environmental tolerance traits. *T. chinensis* plants may also occupy drier habitats in North America than in their native range [4]. Niche shifts to drier habitats could be the result of an altered fundamental niche caused by evolutionary change in the introduced range or a greater range of niche variability in the novel environment [8].

Plants can become tolerant to environmental stress through biochemical and physiological responses resulting from acclimation responses. The molecular, physiological, and transcriptomal responses to drought, low temperature, and salt stress are intertwined [19,20], and many transcription-factor genes (e.g., *DRE*—dehydration responsive element/*CBF-C*-Repeat-Binding Factor; *ERF*—ethylene responsive element; zinc-finger family) that are induced by each stress are similar among stresses [20]. Indeed, DREB1 and CBF are different names for the same transcription factor although the former was isolated under water-stress induction, and the latter, under cold stress. The similarity of responses to these stresses may provide to the plant mechanisms for withstanding each stress singly or in combination, and the ability to grow in a large variety of niche habitats and subsequently be more difficult to control as an invasive plant.

About 35,000 km^2^ (21,000 mi^2^) of habitat is vulnerable to *Tamarix* invasion in Montana, North Dakota and South Dakota [21]. A larger area may be susceptible if conditions become drier and warmer [22,23]. Habitat suitability models predict that croplands and grasslands may be 1.5 to 2 times more likely to support *Tamarix* than wetter areas, where these plants have historically been most successful [24]. There is a need to protect agricultural and natural resources in the United States by preventing *Tamarix* encroachment beyond its current infestation boundaries. However, mechanisms responsible for the success of an invasive plant are difficult to define, and projected climate variability may redefine the parameters of that success [25]. Study of invasive plant transcriptomic responses to abiotic stress offers great potential to uncover critical information about tolerance and adaptation that can hasten early weed detection and control by helping to identify at-risk habitats and informing the management.

The abilities of *Tamarix* to survive drought and salt stress have been used to identify abiotic stress tolerance-related genes. Late embryogenesis abundant (*LEA*) genes from *T. androssowii* confer greater drought tolerance in transgenic poplar (*Populus* sp.) [26] and cold tolerance in transgenic blueberry (*Vaccinium* sp.) [27]. Salt and drought-related *LEA*, dehydration-responsive element binding (*DREB*) genes and other transcription factor genes from *T. androwosii* and *T. hispida* promote salt and water stress tolerance in tobacco (*Nicotiana tabacum*) and thale cress (*Arabidopsis thaliana)* [28,29,30,31,32,33,34,35,36]. While these functional gene analysis studies indicate that there are water and salt stress-associated genes that can contribute to abiotic stress tolerance, transcriptional cataloging has been limited predominately to salt stress transcriptomic profiles in *T. chinensis* and *T. hispida* [31,37]. A recent study presents the diurnal oscillations in gene expression in *T. ramosissima* [38]; however, there is no global transcriptome profiling of water deficit responses in *Tamarix* species or transcriptome comparisons between species. We hypothesize that the drought tolerance responses of *T. chinensis* and *T. ramosissima*, while sharing some characteristics, will have noteworthy differences that contribute to their individual invasive capacity. To facilitate future studies on the invasive character of these species, global transcriptome profiling was conducted in well-watered and water deficit treated seedlings.

## 2. Results

### 2.1. Sequence Quality and de Novo Reference Transcriptome Assembly Show Good Gene Coverage

The Illumina HiSeq2000 yielded a total of 316,308,226 high-quality paired end reads from *T. chinensis* and *T. ramosissima*, which were used to develop a de novo reference *Tamarix* assembly (Table S1). The assembly statistics for the *Tamarix* reference transcriptome are presented in Table 1. Transcriptome analysis for the 1440 benchmarking universal single-copy orthologs (BUSCO), which provides a quantitative assessment of an assembly [39], indicated that 92% of the BUSCO genes were present (419 complete and single copy genes, 777 complete and duplicated genes, and 132 fragmented genes).

### 2.2. Tamarix de Novo Transcriptome Functional Annotation Indicated Strong Homology to Beta vulgaris

**Figure 1:**
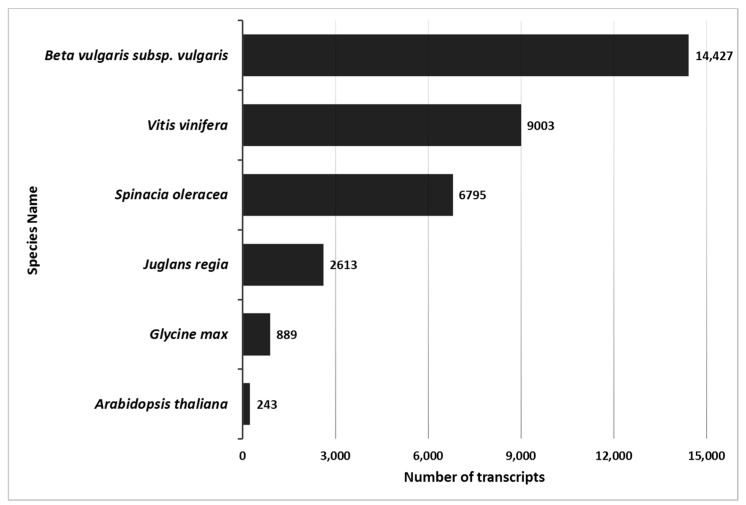
Number of *Tamarix* transcripts matched in other plant species. *Tamarix* transcript matches were identified using BLAT to identify top-hit matches of >95% homology of *Tamarix* contigs with *Beta vulgaris*, *Vitis vinifera*, *Spinacia oleracea*, *Juglans regla*, *Glycine max*, or *Arabidopsis thaliana* transcripts. Specific BLAT settings are noted in the Methods section. The X-axis identifies the number of transcripts, and the Y-axis identifies the name of the species. Numbers next to the bars show the number of homologus transcripts for each species.

Blast2GO analysis of 271,872 transcripts yielded 89,389 homologs to non-redundant eukaryote genes and 68,293 gene ontology terms (GO). The maximum number of homologs was identified for *B. vulgaris.* Both Tamarix and *B. vulgaris* are from the Caryophyllales order. There were fewer homologs identified with other Eudicots (Figure 1, Table S2). The *Tamarix* homologs common with *B. vulgaris* were 450 biological processes GO, 151 cellular component GO, and 462 molecular functions GO.

**Figure 2:**
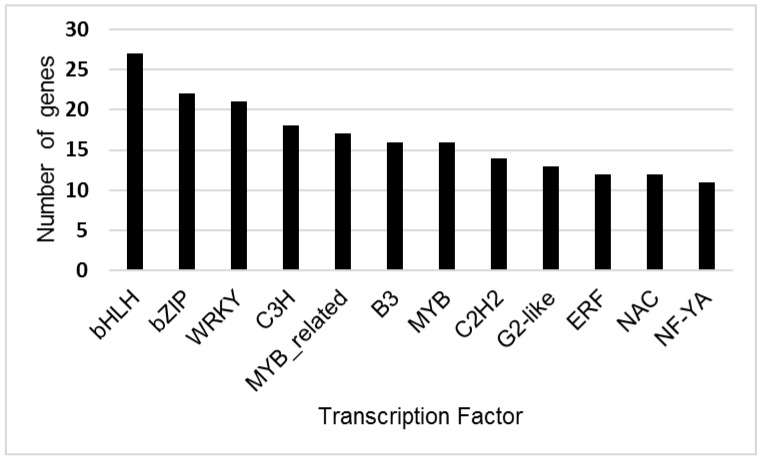
Number of genes identified for *Tamarix* transcription factor families with greater than 10 unigenes present in the transcriptome. Transcription factor genes were predicted from unique *Tamarix* contigs using PLANTTFDB (version 4.0). The X-axis indicates the brief name of the transcription factor family, and the Y-axis shows the number of unique genes identified for each transcription factor family.

CD-Hit analysis identified a total of 198,679 unique transcripts. Prediction of transcription factors for these unique transcripts with PLANTTFDB found 35 transcription factor gene families (Figure 2, Table S3). The largest numbers of unique genes predicted by Blast2GO and Plant TFDB were for the larger transcription factor families, including bHLH, bZIP, WRKY, MYB and NAC.

### 2.3. All Pairwise Comparisons of Species and Water Treatments Show Differential Gene Expression Response Diversity

**Figure 3:**
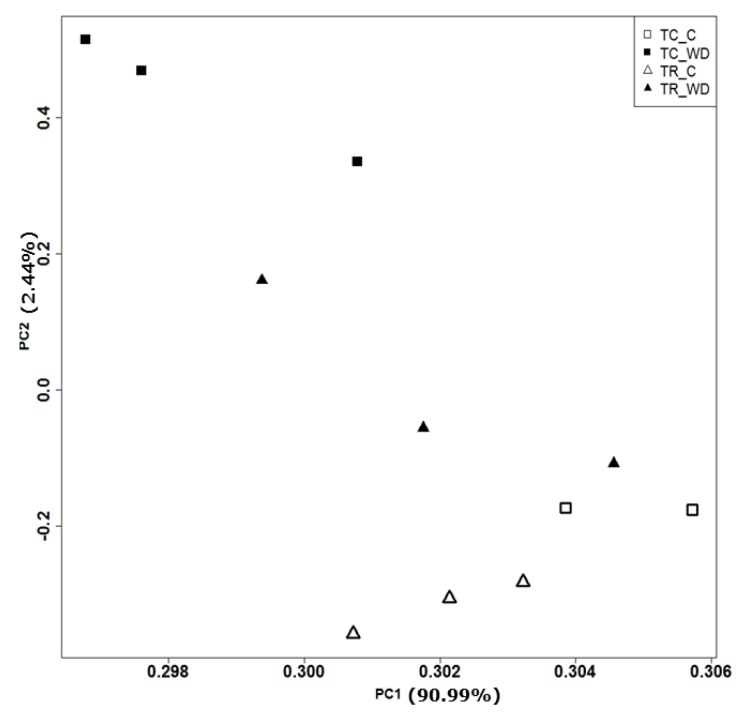
Principal component analysis of *T. chinensis* (TC) and *T. ramosissima* (TR) genes (117,867 genes). Normalized transcript counts for all transcripts were explored using Principal Component Analysis (PCA) in R programming language. Genes are expressed in transcripts per kilobase million (TPM). The open triangles and open squares denote well-watered (C) treatment in TR and TC, respectively. The solid triangles and solid squares denote water deficit (WD) treatment in TR and TC respectively. Percentages of variation explained by PC1 and PC2 were 90.99% and 2.44%, respectively. There are three replicates (*n* = 3) for all treatments except TC_C, where *n* = 2.

**Figure 4:**
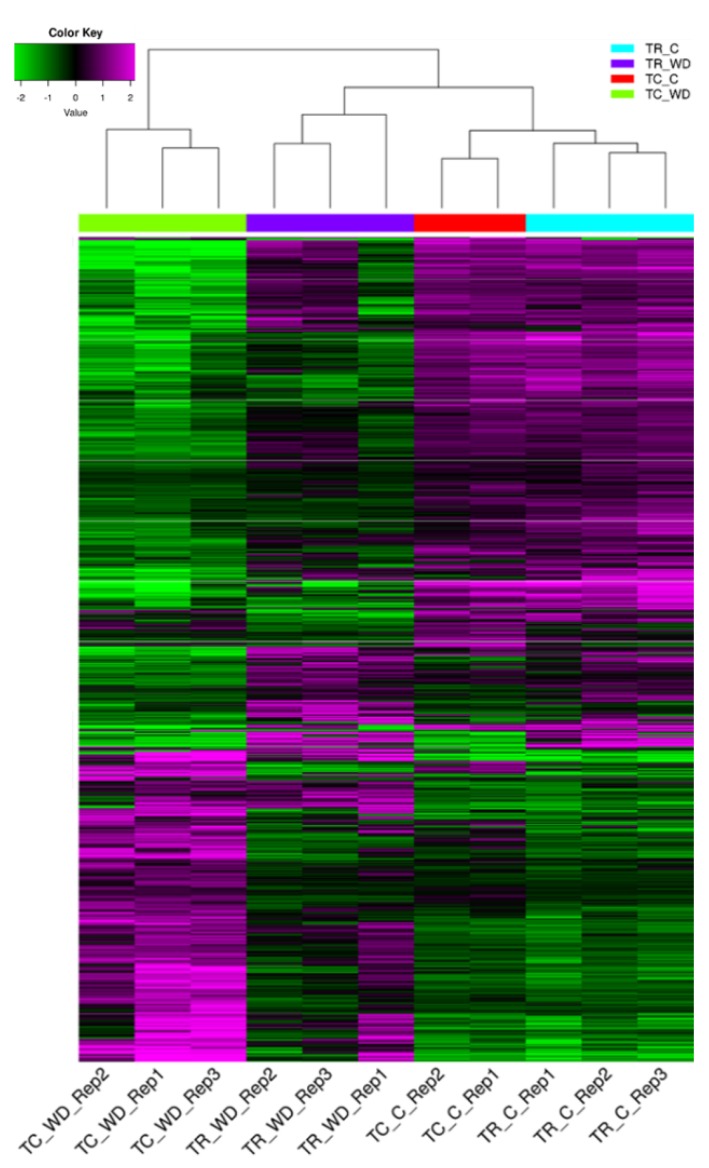
Differentially expressed genes from all pairwise comparisons for *T. chinensis* (TC) and *T. ramosissima* (TR) in well-watered (C) and water deficit (WD) treatments. Differentially expressed values for all genotypes and treatments were determined using a Bioconductor package edgeR (version 3.8.6) with a false discovery rate (FDR) = 0.001 and fold change >2. Magenta color denotes genes up-regulated and green denotes genes down-regulated in WD relative to C in both TC and TR (scale is in the upper left of the figure). Rep1, 2, or 3 denotes the biological replicate number. There are three biological (*n* = 3) replicates in TC_WD, TR_WD, and TR_C, and two biological replicates (*n* = 2) in TC_C.

The principal component analysis (PCA) of 117,867 genes (Table 1, Figure 3) showed a clear distinction between the control and water deficit-treated samples for both species. The PCA explains 93.4% of total variation for species and treatment. There were 3972 differentially expressed genes (DEG) with a false discovery rate (FDR) of 0.001 and log fold change >2 across all pairwise comparisons (six total comparisons) (Figure 4, Table S4). Cluster analysis of the samples indicated that *T. ramosissima* water deficit samples had gene expression responses more similar to the well-watered *T. chinensis* and *T. ramosissima*, although the well-watered controls were the most similar (Figure 4). A group of 2698 genes up-regulated (purple) in *T. chinensis* following drought stress that are not consistently up-regulated in *T. ramosissima* are apparent at the bottom left of Figure 4. Above these are several groups of genes up-regulated in *T. ramosissima* but not in *T. chinensis*.

### 2.4. Differential Gene Expression in Water Deficit Relative to Control Treatment Indicates a Greater Transcriptomic Response in T. chinensis Than in T. ramosissima

**Figure 5:**
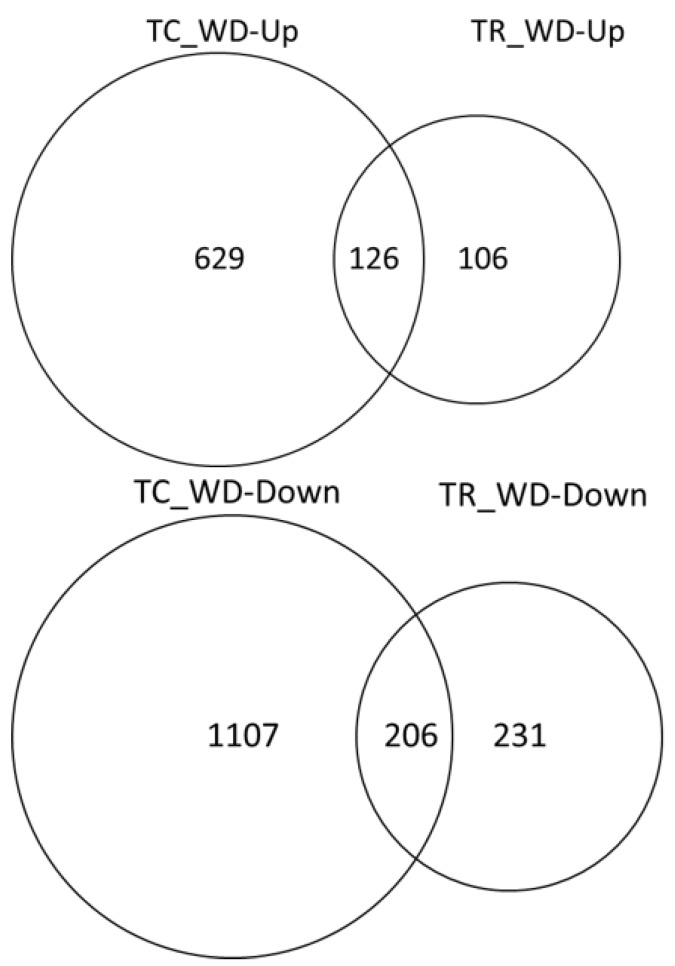
Water deficit response in *T. chinensis* and *T. ramosissima* seedlings. Differentially expressed genes (FDR 0.005 and log fold change ≥1 were identified for TC_well-watered (C) versus TC_water deficit (WD) and TR_C versus TR_WD). *T. chinensis* water deficit up- or down-regulated (TC_WD-Up or -Down) and *T. ramosissima* (TR_ WD-Up or -Down) indicate the number of differentially expressed genes that are up- or down-regulated in the water deficit treatment (WD) relative to the well-watered control (C).

About twice as many DEG were down-regulated under water deficit conditions relative to the well-watered conditions in both species (Figure 5). There were a greater number of unique DEG in water deficit samples relative to their well-watered control in *T. chinensis* (1736) than in *T. ramosissima* (337) (Figure 5). In addition, there were 332 DEG in common between the two species.

### 2.5. Gene Ontology Classification Identified Similar Functional Categories for T. chinensis and T. ramosissima

Gene ontology term (GO) classification of DEG up-regulated in water deficit treatment relative to control for *T. chinensis* and *T. ramosissima* indicated similar GO sub-categories of cellular components. In the cellular component, GO categories indicated that there was a greater proportion of membrane and membrane part genes in *T. ramosissima* than in *T. chinensis* (Table S5). The largest proportion of molecular function GO categories were catalytic activity and binding categories, and there was a larger percentage of genes in *T. chinensis* compared to *T. ramosissima*.

**Figure 6:**
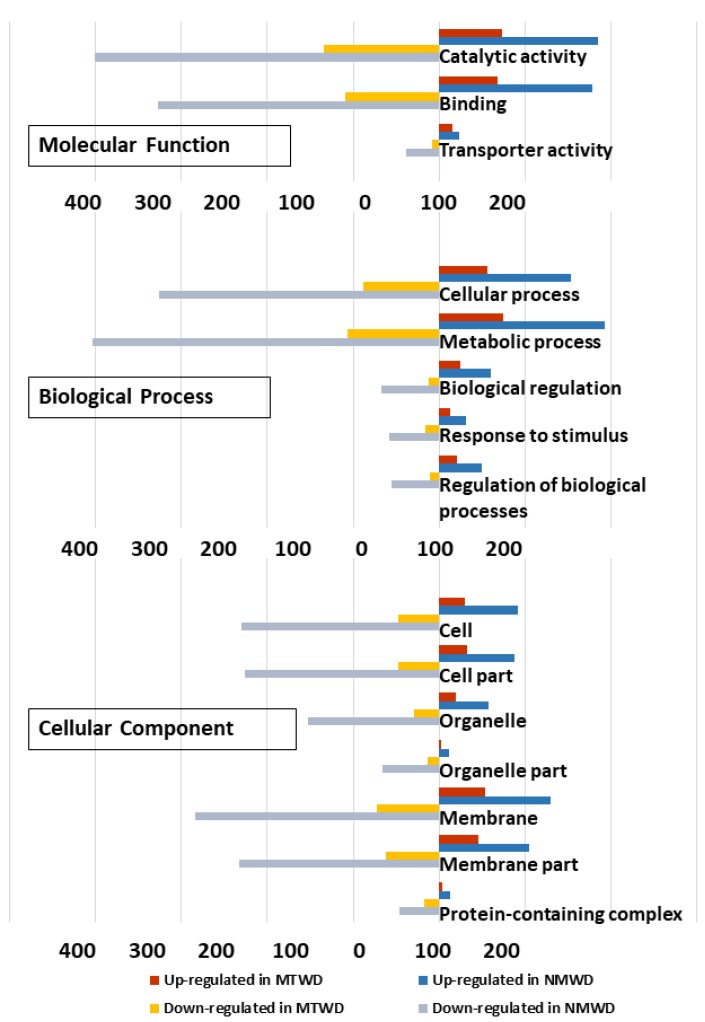
Gene ontology classification of differentially expressed genes in *T. chinensis* (TC) and *T. ramosissima* (TR). Differentially expressed gene ontology terms were functionally classified and visualized with Web Gene Ontology Annotation Plot (WEGO, version 2.0). Blue and red bars represent the number of genes up-regulated in TC_water deficit treatment (WD) and TR_WD relative to their respective well-watered control. Gray and yellow bars represent the number of down-regulated genes in TC_WD and TR_WD relative to their respective well-watered control.

The biological processes category contained the largest number of genes (Table S5). The major categories in common between species were cellular process, metabolic process, biological regulation, response to stimulus and regulation of biological processes, and they showed a similar proportion of differentially up- and down-regulated genes in each species (Figure 6).

In both species, there was a greater down-regulation across GO categories than there was up-regulation (Figure 6, Table S5). However, there was a greater percent of significantly enriched and down-regulated in the transporter activity category for *T. chinensis* than in *T. ramosissima.* In contrast, a greater percentage of catalytic activity and binding in the molecular function categories were significantly enriched for *T. ramosissima* (Figure 6, Table S5).

### 2.6. Tamarix Homologs with B. vulgaris and A. thaliana

*Tamarix* gene homologs with *B. vulgaris* and *A. thaliana* genes were significantly enriched in biological processes, cellular component and molecular function gene ontology categories and KEGG pathways. *Tamarix* homologs with *A. thaliana* were found to be signficantly enriched in KEGG pathways such as oxidative phosphorylation, MAPK signaling pathway, Phosphatidylinositol signaling system, and MRNA surveillance pathway (Tables S6–S8).

### 2.7. Biological Processes and Molecular Function Networks Indicated Distinct Differences in the Water Deficit Responses of T. chinensis and T. ramosissima

**Figure 7:**
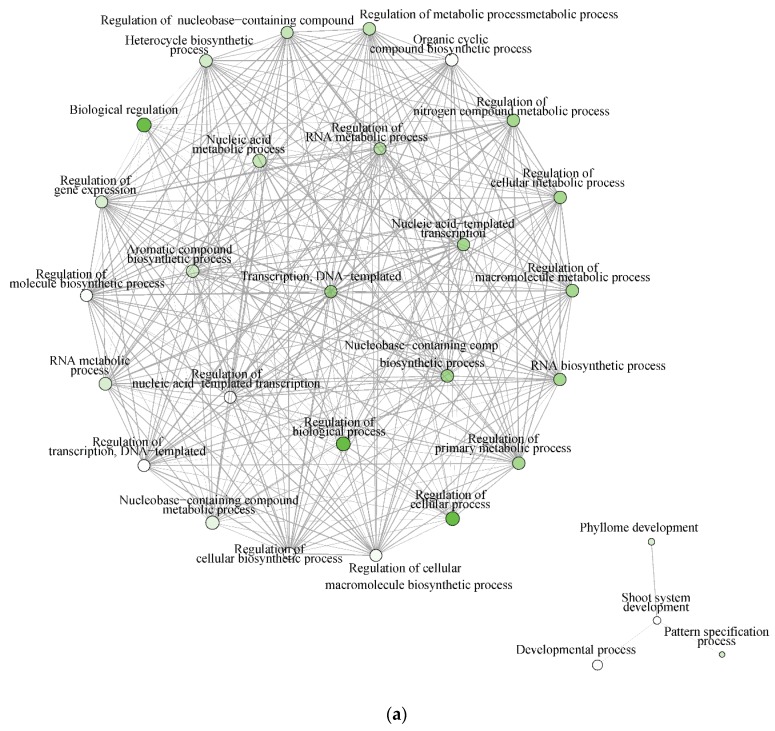

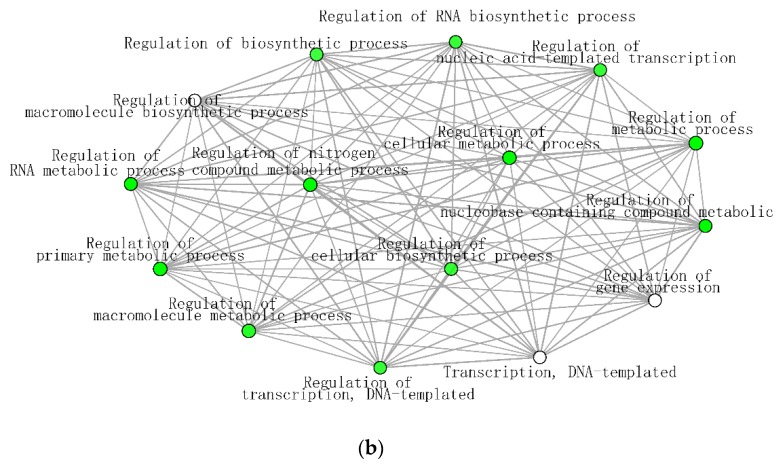
(**a**) Significantly enriched biological processes for *T. chinensis* genes up-regulated under water deficit treatment (TCWD) relative to its well-watered control. Significant enrichment networks were performed using DEG in the iGraph R package in the ShinyGO web server. Networks consist of nodes and lines connecting the nodes. Nodes are circles, which denote the different biological process categories. The color of the circle denotes the direction of regulation of the genes belonging to each enriched biological process in the network. Lines denote the interactions, which are the genes that are common between any two biological processes. Darker green color in the circles indicates greater up-regulation of genes. (**b**) Significantly enriched biological processes for *T. ramosissima* genes up-regulated under water deficit treatment (TRWD) relative to its well-watered control. Significant enrichment networks were performed using DEG in the iGraph R package in the ShinyGO web server. Network consists of nodes and lines connecting the nodes. Nodes are circles, which denote the different biological process categories. The color of the circle denotes the direction of regulation of the genes belonging to each enriched biological process in the network. Lines denote the interactions, which are the genes that are common between any two biological processes. Green color in the circles indicates greater up-regulation of genes. Enriched GO categories down-regulated in TRWD were negative regulation functional categories (cellular processes, gene expression, signaling and RNA biosynthesis) and functional categories related to the positive regulation of chromatin and histone modifications (Table S6d).

The significantly enriched biological processes for *T. chinensis* and *T. ramosissima* water deficit treatments (TCWD and TRWD) each show a large network of >70 genes and 68 genes up-regulated in WD relative to well-watered control in-common for the two species. These networks include 30 biological processes functional categories, of which eight are common between species (Figure 7a,b). The categories that are in common account for 27% and 40% of the DEG identified in TCWD and TRWD, respectively. TCWD had unique enriched functional categories related to regulation of gene expression, RNA and DNA transcription, and biosynthesis and metabolic processes. TRWD showed enrichment in biological functional GO categories related to signaling and response to stimulus, as well as clock-related categories (Table S6a,b). Enriched biological processes functional GO categories down-regulated in both TCWD and TRWD included several cellular biosynthetic processes (Figure S1a,b; Table S6c,d). The GO categories down-regulated in common account for 22% and 39% of the DEG identified in TCWD and TRWD, respectively. The functional GO categories uniquely down-regulated in TCWD included regulation of many primary and secondary biosynthetic and metabolic categories (Table S6c). It is also of note that cellular component GO categories that were down-regulated in TCWD were predominately photosystem-related.

There were only four molecular function GO categories up-regulated in TRWD, and they were in common with TCWD. TCWD had 26 enriched molecular function categories for the up-regulated DEG that were clustered into two networks. One was related to transcription and binding categories and the other related to kinase, phosphotransferase, and ATP and nucleotide binding (Figure S2a, Table S7a). There was one small network of transcription and binding GO categories up-regulated in TRWD (Table S7b). Two down-regulated networks were identified for TCWD (Figure S2b, Table S7c). There were no molecular function networks identified for the down-regulated DEG in TRWD (Table S7d). Up-regulated cellular components GO categories in TCWD were photosynthetic-related (Table S7e).

### 2.8. Unique Transcription Factors Are DEG in T. chinensis and T. ramosissima in Response to Water Deficit

Several drought-related *Arabidopsis* transcription factor homologs are up-regulated in both species (Table S8). This includes *ABA* 8′-hydroxylase (*CYP707a3)*, myb domain protein (*MYB 27*, *106*), NAC domain containing protein (*NAC 103*) transcription factor, dehydrin family protein (*RAB18*), peroxidase superfamily protein, and galactose oxidase/kelch repeat superfamily protein. Similarly, a *NAC* homolog for *Vitis vinifera NAC 47* was up-regulated in both TCWD and TRWD. Transcription factor genes uniquely up-regulated in TCWD included MADS-box transcription factor family protein (*SHP1*); MYB *23*, *31*, *59*, *92*, *120*; WRKY DNA-binding protein (*WRKY 18*, *26*); Wuschel-related homeobox 12 (*WOX12*); and YABBY family protein (*YAB5*). Water deficit-related genes uniquely up-regulated in TRWD included the ethylene-related genes (ethylene-forming enzyme (*EFE*); *NAC076* and *ERF/AP2* transcription factor family genes).

There were 10,401 homologs to *Vitis vinifera* genes in the reference *Tamarix* transcriptome. These matched 254 and 71 of the TCWD and TRWD DEG, respectively. VitisNet Pathway enrichment analysis of the TCWD and TRWD DEG showed positive enrichment of the WRKY transcription factors with WRKY 57 homolog in both species. In addition, genes related to starch and sucrose metabolism and ethylene response factor transcription factors (*ERF1*, *SHINE 3*) were enriched in TCWD but not in TRWD (Table S8).

## 3. Discussion

### 3.1. Water Deficit Leaf Reference Transcriptome Shows a Greater Number of Genes Than Salt Stressed Roots

*Tamarix* species, commonly referred to as tamarisk, are invasive species in the United States. Since *T. chinensis* introduction in the 19th century and other species in the 20th century [2], *Tamarix* species and hybrids now extend from the southwest to the north-central region. *Tamarix* is a weed management issue; even young plants (12 weeks old) are difficult to control, as greater than 30% of *T. chinensis* and *T. ramosissima* plants are shown to survive fire and mowing treatments [40]. *T. chinensis* and *T. ramosissima* are more drought tolerant than many native riparian species, contributing to the invasive success [41,42,43,44]. *T. ramosissima* is noted to tolerate more water stress than native phreatophytes and maintains its growth capacity during periodic drought conditions, allowing it to dominate and create high-density stands in the floodplain and arid region communities in the Mojave Desert (Nevada, USA) [41]. It is noteworthy that *T. ramosissima* took seven days longer to show leaf wilt than *T. chinensis* in this study.

There is currently no published genome for any *Tamarix* species; therefore, a reference transcriptome was assembled using the combined RNAseq data from both species. The reference transcriptome of *T. chinensis* and *T. ramosissima* yielded a robust 117,867 unigenes. Benchmarking of the reference transcriptome indicated the presence of 92% of the universal single-copy orthologs [39]. The reference transcriptome described herein had a greater number of unigenes than that found in salt stressed roots (47,000 to 59,000 (*T. hispida* and *T. chinensis*)) or in the diurnal transcriptome of leaves (72,035, *T. ramosissima*) [31,37,38]. Functional gene ontology term classification of the DEG within molecular function, biological processes and cellular component GO categories indicated that the functional GO categories with the greatest number of DEG were similar to those found under salt stress for *T. hispida* and *T. chinensis* roots [31,37].

There is currently no published genome for any *Tamarix* species; therefore, analyses of the de novo reference transcriptome was conducted using *Arabidopsis thaliana*, other woody species and two domesticated species of the order Caryophylalles (*Spinacia oleracea* [spinach] and *B. vulgaris* [sugar beet]) [45,46,47]. *Tamarix* showed the greatest homology to the double haploid sugar beet line KWS2320, with over 14,000 homologs.

### 3.2. Differential Gene Expression Differs in Response to Water Deficit in T. chinensis and T. ramosissima

Differential gene expression analysis identified distinctly different expression patterns between the two species. There were 126 DEG up-regulated in WD relative to well-watered C and 206 DEG down-regulated in WD relative to well-watered C in common between *T. chinensis* and *T. ramosissima*. Top hits of DEG up-regulated in common under deficit treatments included known drought-responsive genes. Previous studies in salt, osmotic, metal and water deficit stress identify *NAC*, *bHLH*, *MYB WRKY* and *ERF* transcription factors and dehydrin or late embryogenesis abundant (*LEA*) genes as having a role in stress tolerance in *Tamarix* species [29,30,31,32,35,48,49,50,51].

Dehydrin/LEA proteins play a role in drought and salt stress, and 21 *LEA* genes are up-regulated in salt-stressed *T. hispida* [32,36]. A *LEA* gene from *T. androssowii* improves salt and drought tolerance in transgenic tobacco and poplar [26,35,52]. A *T. hispida* dehydration-responsive element-binding (*DREB*) gene increases the salt and drought tolerance of transgenic tobacco and *T. hispida* [53]. It is of note that a dehydrin family protein (*RAB18*) was up-regulated in both *T. chinensis* and *T. ramosissima* during water deficit, suggesting a potential role in water stress tolerance.

In *T. hispida*, fourteen *MYB* transcription factors were characterized under osmotic and salt stress and *MYB13* was induced during salt stress [50]. Over-expression of *MYB13* in *T. hispida* resulted in low expression of reactive oxygen species, and knockout of *MYB13* had the opposite effect, suggesting that *MYB13* played a role in salt tolerance by regulating reactive oxygen species. In the current study, *MYB27* and *MYB106* were up-regulated in both *T. chinensis* and *T. ramosissima*, indicating these genes as having a potential role in water deficit response that should be explored. Similarly, over-expression of a *T. hispida* basic helix-loop-helix (*bHLH*) leucine-zipper transcription factor in *Arabidopsis* increases expression of *LEA* and reactive oxygen scavenging (*ROS*) genes and ROS enzyme activity, enhancing salt and osmotic-stress tolerance [29]. In the present study however, six *bHLH* genes were up-regulated in *T. chinensis* but none in *T. ramosissima*.

Ethylene response factors (*ERF*) are expressed in the root, stem and leaves of *T. hispida* in response to salt and drought stress, and *ERF1* is most abundant, suggesting a potential role in stress tolerance [48]. In contrast, over-expression of *T. hispida ERF1* led to increased abiotic stress sensitivity, suggesting a negative regulation of reactive oxygen scavenging ability [32]. In the current study, *ERF1* is up-regulated in both species; however, different peroxidase genes are up- and down-regulated, suggesting that there may be negative regulation of specific reactive oxygen scavenging-related genes. In *Arabidopsis*, down-regulation of *ERF1* occurs in response to overexpression of a *DREB* gene and is associated with an increase in *WRKY* gene expression and drought tolerance [53]. Similarly, over-expression of a *T. hispida WRKY* gene increased the salt and oxidative stress tolerance of *Arabidopsis* by increasing the activity of super-oxide dismutase and peroxidase genes and decreasing levels of reactive oxygen species [50]. The *WRKY* genes (18 and 26) are up-regulated in *T. chinensis*, and *WRKY57* is up-regulated in both *T. chinensis* and *T. ramosissima. WRKY57* was also up-regulated in the woody liana *Vitis riparia* during water deficit, which is a strong potential indication of a role for the *WRKY57* transcription factor in water deficit response [54].

In *T. hispida*, 21 *NAC* genes are up-regulated in response to salinity, drought, heavy metals and abscisic acid, indicating a role in abiotic stress tolerance [32]. *NAC13* increases salt and osmotic stress tolerance in transgenic *Arabidopsis* and *Tamarix*, promoting root growth and increased superoxide dismutase and peroxidase activities [37]. In this study, *NAC102* and *NAC76* were up-regulated in *T. chinensis* and *T. ramosissima*, respectively. However, *NAC103* and *NAC47* and peroxidase genes were up-regulated in both *T. chinensis* and *T. ramosissima* in response to water deficit. In *V. riparia*, *NAC47* and peroxidase and superoxide dismutase genes are also up-regulated in leaves in response to water deficit [54]. This suggests a potential role for *NAC47* and *NAC103* and peroxidase regulation in water deficit tolerance [37].

*T. chinensis* had a greater change in gene expression in response to water deficit than *T. ramosissima* did. The common expression of the transcription factors *MYB27*, *MYB106*, *NAC47*, *NAC103*, and *WRKY57* in *T. chinensis* and *T. ramosissima* suggests a strong potential role in water deficit tolerance in *Tamarix* that should be explored relative to the regulation of reactive oxygen scavenging genes. Dehydrin/LEA protein genes were up-regulated in *T. chinensis* and *T. ramosissima*, suggesting potential for increased drought tolerance. Up-regulation of *ERF1* in *T. chinensis* and *T. ramosissima* and up- and down-regulation in different peroxidase genes also shows how both species differentially modify transcriptomic responses during drought stress. GO enrichment analysis of TCWD up-regulated DEG showed enriched biological functional categories related to regulation of gene expression, RNA and DNA transcription, biosynthesis and metabolic processes. In contrast, TRWD up-regulated DEG were more related to an early gene expression cascade response as the DEG included GO enrichment in signaling and response to stimulus, and in clock-related categories. Enriched GO biological process categories for TCWD down-regulated DEG were many primary and secondary biosynthetic and metabolic processes, and enriched GO cellular component categories were photosystem-related. Enriched categories for the TRWD down-regulated DEG included cellular processes, gene expression, signaling and RNA biosynthesis, and positive regulation of chromatin and histone modifications. The TCWD and TRWD DEG were also enriched in the WRKY pathway as indicated by homolog analysis in the VitisNet pathways.

We found distinct differential gene expressions in *T. chinensis* and *T. ramosissima* (typically found in the southwest United States and in more northern latitudes, respectively) in flaccid leaves in response to water deficit. While *Tamarix* spp. are not known for water use efficiency (WUE), *T. ramosissima* appears to have better WUE than *T. chinensis* [41]. In the fourteen week-old plants in this study, *T. chinensis* reached leaf wilt sooner than *T. ramosissima* did (10 and 17 days, respectively). Differential gene expression analysis indicated that *T. chinensis* had more down-regulated genes and pathways than *T. ramosissima*. The ability to withstand drought may help maintain growth capacity during dry periods and permit a faster recovery when water is available. Since genes expressed under drought and cold stress are similar, better drought tolerance also may explain better tolerance to cold and salt stress [42,43]. Indeed, *T. ramosissima* is reported in more northern habitats (up to 53° N in Asia) [55]. Greater drought tolerance may also have implications for herbicide tolerance by lessening uptake during dry conditions, with plants having closed stomata or a thicker waxy cuticle. In addition, MYB27 and MYB106 were up-regulated in both species under drought, increasing reactive oxygen scavenging and potentially enhancing salt and osmotic stress tolerance, making these species potentially more resilient to multiple stress factors than any native competing species. These findings may also have implications in the search for drought tolerant genes for commercial crops, by finding genes that could enhance or maintain growth during periodic seasonal dry conditions.

## 4. Materials and Methods

### 4.1. Plant Materials

Seeds of *T. chinensis* were obtained from New Mexico (Latitude 32.40° N, Longitude 104.20° W) and *T. ramosissima* from Montana (Latitude 47.60° N, Longitude 107.42° W) from established stands on 5 August and 9 October 2013, respectively. Seeds were stored in a porous container at 5 °C until planting to maintain viability.

*T. chinensis* and *T. ramosissima* seeds were planted on 11 October 2013 on the surface of Ray Leach SC10 Super cone-tainer cells (Stuewe and Sons, Inc., Corvallis, OR, USA) (3.8 cm [1.5 in] diameter by 21 cm [8.3 in] length) filled with sandy clay loam topsoil (collected from Brookings, SD) that had been passed through a 2 mm sieve. Cone-tainers were stabilized using support racks and placed inside plastic tubs filled with water (to 15 cm) to maintain surface wetness and maximize germination and seedling growth [40]. Tubs were placed in a greenhouse with natural and artificial lighting (12/12 h light/dark cycle), and temperatures ranged between 20 and 30 °C. Plants were thinned to one cone^−1^ about 3 weeks after germination, and water levels were allowed to drop to 10 cm.

### 4.2. Genotype Analysis

Leaf tissue was collected from ten individuals grown from the *T. chinensis* and *T. ramosissima* on 27 January 2014. *Tamarix* species identities were determined through Amplified Fragment Length Polymorphism (AFLP) analyses performed at the USDA Northern Plains Agricultural Research Laboratory in Sidney, MT, USA [56].

### 4.3. Water Deficit Treatment

On 17 January 2014, fourteen week-old plants were placed in growth chambers maintained at 25 °C 12/12 h light/dark cycle. Plants were subjected to well-watered control (C) (sub-irrigated in tubs with water depth of 3 cm) or water deficit treatments. Water deficit (WD) was imposed by withholding water until water stress was shown by flaccid/wilting leaves.

*T. chinensis* and *T. ramosissima* reached wilting point at 10 and 17 days, respectively, after water was withheld. At the wilting point, all leaf tissue was collected from three separate replicates (one plant per replicate sample), which were sampled for each of the WD and well-watered control treatments for each species, and was immediately submerged in liquid N and stored at −80 °C for subsequent RNA extraction.

### 4.4. RNA Extraction

A modified protocol using the Sigma Spectrum Plant Total RNA isolation kit was developed for efficient *Tamarix* RNA isolation. A 100 mg sample of leaf tissue was finely ground to a talc-like powder in liquid nitrogen, and then 800 µL lysis buffer was added and homogenized by grinding until completely thawed. The mixture was transferred to a 2 mL collection tube, incubated at room temperature for 10 min, and centrifuged at 9000 rpm for 4 min to pellet the cellular debris. A 700 µL aliquot of the supernatant was pipetted (without disturbing the debris pellet) into a 1 mL filtration column placed in a 2 mL collection tube. The supernatant and filtration column were centrifuged for 1 min at maximum speed, the column was discarded, and the clarified lysate saved. Binding solution (500 µL) was added to samples and mixed well by vortexing quickly or pipetting back and forth. A 750 µL aliquot of this mixture was transferred into a binding column, centrifuged for 1 min at maximum speed, flow-through discarded, and steps repeated for the remaining mixture. The column was placed into a new 2 mL collection tube, 550 µL of wash Solution 1 was added to the column and centrifuged for 1 min at maximum speed, and the flow-through was discarded. The column was placed into a new 2 mL collection tube. Diluted Wash Solution 2 (550 µL) was added to the column and centrifuged for 30 s at maximum speed. This was repeated once more. The flow-through was discarded each time. The column was placed in a new 2 mL collection tube and centrifuged for 1 min at maximum speed to dry. The column was transferred to a new, clean 2 mL tube, 40 µL of elution solution was placed onto the center of the binding matrix, and it was incubated for 1 min. The tube was then centrifuged for 1 min at maximum speed to elute the RNA. The column was discarded, and the eluate containing total RNA was assayed for quantity and quality. The absorbance ratios at 260 to 280 nm and at 260 to 230 nm, and RNA yield were obtained using a NanoDrop ND-1000 spectrophotometer (Thermo Fisher Scientific, Wilmington, DE, USA). RNA quality was checked using an Agilent 2100 Bioanalyzer (Agilent Technologies Inc., Santa Clara, CA, USA).

Leaf tissue of *T. chinensis* and *T. ramosissima* at wilting point, i.e., 10 and 17 days, respectively, was collected from three biological replicates of the WD and well-watered control treatments for each species. Samples were sent to BGI (BGI@ UC Davis Genome Center, Sacramento, CA, USA) for cDNA library preparation and sequencing. NEBNext Ultra RNA Library Prep kits were used for cDNA library construction, and cDNA libraries were sequenced using a paired end protocol on an Illumina HiSeq2000 at BGI, University of California Davis Genome center, Sacramento, CA. Raw sequence reads were paired end and 100 base pairs long for each replicate sample. One *T. chinensis* C sample was lost in the transfer process, resulting in 2 replicates being sequenced.

### 4.5. De Novo Reference Transcriptome Assembly and Assessment

Raw sequence reads were checked for quality with FastQC (version 0.11.3) [57]. Reads were trimmed from 5′ and 3′ ends to a Phred quality score of ≥20 with Prinseq (version 0.20.4) [58], and only reads with more than 20 nucleotides were maintained for transcriptome assembly and gene expression analysis.

A de novo reference *Tamarix* transcriptome was assembled using quality reads with Trinity software (version 2.4.0) [59] and paired end script with a default kmer of 25. The basic statistics of the de novo transcriptome were generated using Trinity software stats.pl script. BUSCO (Benchmarking Universal Single-Copy Orthologs) (version 3) [39] was employed to test the relative completeness of the reference transcriptome using a Eukaryotic lineage dataset; embryophyte_odb9, and 1440 BUSCO groups, with *A. thaliana* as the Augustus default species.

### 4.6. Functional Gene Annotation

Blast2GO (version 4.1.9) [60,61] was used to functionally annotate the *Tamarix* contigs. The de novo contigs were used as query sequences against a non-redundant eukaryotic protein database using CloudBlast with parameters; BLASTX, a word size of 3, an expectation value of 1 × 10^−10^ a maximum target hit sequence of 1, and a Highest Scoring Pair (HSP) length cutoff of 33. The top-hit homologs were identified with a BLASTX search. GO [62,63,64] and GO Consortium 2019 terms for these top-hit homologs were retrieved using Blast2GO (version 4.1.9).

Unique contigs were identified using Cd-Hit (version 4.6.7) [65]. Transcription factors were predicted from the unique *Tamarix* contigs using PLANTTFDB (version 4.0) [66,67]. Transcription factors predicted using PLANTTFDB were compared with the transcription factors obtained with Blast2GO.

### 4.7. GO and KEGG Pathway Enrichment

The cDNA sequences of *A. thaliana* [68], *B. vulgaris* [69] and *V. vinifera* version1 genome [70] were used in a BLAT (version 351) [71] search for top-hit matches of >95% identity against the *Tamarix* contigs. Other parameters used in BLAT were tile size of 11 for DNA sequences, no. of tile matches of 2 for nucleotide sequences, minimum score of 20, maximum gap between tiles as 2, and maximum intron size of 750,000. Pathway and gene ontology enrichment was conducted for the *B. vulgaris* (Refbeet 1.2.2) [45] and *A. thaliana* homologs using ShinyGO [72].

### 4.8. Gene Expression Analysis

The RSEM (RNA-Seq by Expectation Maximization) software package [73] was used to align the cleaned raw sequence reads against the reference *Tamarix* contigs with bowtie aligner (version 2) [74,75] (using the scripts align_and_estimate_abundance.pl and abundance_estimates_to_matrix.pl) to estimate the number of fragments that map to each contig. Normalized expression levels in TPM (transcripts per million), FPKM (fragments per kilobase per million mapped reads) and counts matrix for the control and treatment samples were obtained. Normalized transcript counts for all transcripts were explored using Principal Component Analysis (PCA) in R programming language.

### 4.9. Pairwise Comparisons of All Species and Water Treatments

Differential expression was determined using a Bioconductor package edgeR (empirical analysis of DGE in R) (using the script run_DE_analysis.pl) (version 3.8.6) [76]. Differentially expressed genes (DEG) were defined as those expressed with |log fold change ≥2| and false discovery rate (FDR) of 0.001. Differential expression in *T. chinensis* and *T. ramosissima* and control and water deficit treatment samples were visualized with iDEP (version 0.71) [77] using TPM above zero in at least one sample, and hierarchical clustering with parameters; Euclidean distance and average linkage.

### 4.10. Differential Gene Expression in Water Deficit Relative to Control Treatment

Differentially expressed genes (FDR 0.005 and |log fold change ≥1|) were identified with pairwise comparisons (TC_C Vs TC_WD and TR_C Vs TR_WD). DEG GO terms were functionally classified and visualized with Web Gene Ontology Annotation Plot (WEGO, version 2.0) [78,79]. The DEG were mapped to their *A. thaliana* homologs (Figure 5). These homologs were tested for their enrichment in GO biological process, GO cellular component, GO molecular function and KEGG pathways with ShinyGO [72]. The input of ShinyGO is the differentially expressed genes list, and the application performs gene ontology analysis based on Ensembl v92 (over 200 plant and animal species). The significantly enriched biological processes/molecular functions/cellular components/kegg pathways were identified using a hypergeometric test, and Bonferroni correction was applied to adjust the false discovery rate (FDR), as described in ShinyGO [72]. Network enrichment analyses were performed for the TCWD and TRWD DEG using the iGraph R package in the ShinyGO web server [72]. Related GO terms are connected by a line, whose thickness reflect the percent of overlapping genes. Size of the node corresponds to number of genes. The DEG were also mapped to their *V. vinifera* homologs and tested for pathways using VitisNet pathways [80,81,82]. Significantly enriched VitisNet pathways were obtained with the VitisPathways tool using 1000 permutations and a Fisher’s exact test with *p*-value < 0.05 and permuted *p*-value < 0.05 [80,81,82].

### 4.11. Data Availability

The RNA-seq data for this study have been deposited into the National Center for Biotechnology Information GEO database under accession GSE127198.

## Figures and Tables

**Table 1:** Tamarix de novo reference transcriptome assembly statistics using a Trinity assembler.

Description	Number
Total genes according to Trinity Assembly	117,867
Total Trinity transcripts	271,872
Percent Guanine-Cytosine	40.9
Contig N10	4150
Contig N20	3234
Contig N30	2659
Contig N40	2215
Contig N50	1824
Median contig length (bp)	622
Average contig length (bp)	1058
Total assembled bases	287,833,633

## Supplementary Materials

The following are available online at https://www.mdpi.com/2223-7747/9/1/86/s1, Figure S1: (**a**) Significantly enriched biological processes for *T. chinensis* genes down-regulated under water deficit treatment (TCWD) relative to its well-watered control. (**b**) Significantly enriched biological processes for *T. ramosissima* genes down-regulated under water deficit treatment (TRWD) relative to its well-watered control. Significant enrichment networks were performed using DEG in the iGraph R package in the ShinyGO web server. Network consists of nodes and lines connecting the nodes. Nodes are circles, which denote the different biological process categories. Lines denote the interactions, which are the genes that are common between any two biological processes. Green color in the circles indicates greater down-regulation of genes. Figure S2: (**a**) Significantly enriched molecular function categories up-regulated in *T. chinensis* under water deficit treatment (TCWD) relative to its well-watered control. (**b**) Significantly enriched molecular function categories down-regulated in *T. chinensis* under water deficit treatment (TRWD) relative to its well-watered control. Significant enrichment networks were performed using DEG in the iGraph R package in the ShinyGO web server. Network consists of nodes and lines connecting the nodes. Nodes are circles, which denote the different biological process categories. Lines denote the interactions, which are the genes that are common between any two biological processes. Table S1–S4: S1. Summary of Raw and clean paired end sequence reads. S2. Summary of number of Blast2GO hits for *Tamarix* transcripts. S3. *Tamarix* transcription factor gene families. S4. Differentially expressed genes in *Tamarix chinensis* and *Tamarix ramosissima.* Table S5: Gene ontology classification of differentially expressed genes in *T. chinensis* and *T. ramosisima*. Table S6: Biological processes up- and down-regulated in *T. chinensis* and *T. ramosissima.* a. Enriched biological process categories up-regulated in *T. chinensis*. b. Enriched biological process categories up-regulated in *T. ramosissima*. c. Enriched biological process categories down-regulated in *T. chinensis*. d. Enriched biological process categories down-regulated in *T. ramosissima*. Table S7: Molecular function categories up- and down-regulated in *T. chinensis* and *T. ramosissima.* a. Enriched molecular function categories up-regulated in *T. chinensis*. b. Enriched molecular function categories up-regulated in *T. ramosissima*. c. Enriched molecular function categories down-regulated in *T. chinensis*. d. Enriched biological processes down-regulated in *T. ramosissima*. e. Enriched cellular component categories down-regulated in *T. chinensis.* Table S8: *Arabidopsis* and *Vitis* homologs for differentially expressed gene. S8a. *Arabidopsis* and *Vitis* homologs for DEG up-regulated in *T. chinensis* water deficit relative to control. S8b. *Arabidopsis* and *Vitis* homologs for DEG up-regulated in *T. ramosissima* water deficit relative to control.
